# Photoacclimation of the polar diatom *Chaetoceros neogracilis* at low temperature

**DOI:** 10.1371/journal.pone.0272822

**Published:** 2022-09-20

**Authors:** Thomas Lacour, Jade Larivière, Joannie Ferland, Philippe-Israël Morin, Pierre-Luc Grondin, Natalie Donaher, Amanda Cockshutt, Douglas A. Campbell, Marcel Babin

**Affiliations:** 1 Ifremer, PHYTOX, PHYSALG, Brest, France; 2 Département de Biologie, Takuvik International Research Laboratory (IRL-3376, CNRS (France) & ULaval (Canada), Université Laval, Québec, Canada; 3 Department of Chemistry and Biochemistry, Mount Allison University, Sackville, Canada; Texas A&M University at Galveston, UNITED STATES

## Abstract

Polar microalgae face two major challenges: 1- growing at temperatures (-1.7 to 5°C) that limit enzyme kinetics; and 2- surviving and exploiting a wide range of irradiance. The objective of this study is to understand the adaptation of an Arctic diatom to its environment by studying its ability to acclimate to changes in light and temperature. We acclimated the polar diatom *Chaetoceros neogracilis* to various light levels at two different temperatures and studied its growth and photosynthetic properties using semi-continuous cultures. Rubisco content was high, to compensate for low catalytic rates, but did not change detectably with growth temperature. Contrary to what is observed in temperate species, in *C*. *neogracilis*, carbon fixation rate (20 min ^14^C incorporation) equaled net growth rate (μ) suggesting very low or very rapid (<20 min) re-oxidation of the newly fixed carbon. The comparison of saturation irradiances for electron transport, oxygen net production and carbon fixation revealed alternative electron pathways that could provide energy and reducing power to the cell without consuming organic carbon which is a very limiting product at low temperatures. High protein contents, low re-oxidation of newly fixed carbon and the use of electron pathways alternative to carbon fixation may be important characteristics allowing efficient growth under those extreme environmental conditions.

## 1. Introduction

Most of polar phytoplankton species have optimum growth temperatures <10°C and upper limits <15°C [1]. The optimal temperature for growth of a microalgae is strongly related to the mean annual temperature at the isolation location, suggesting major adaptations to local environmental conditions [1]. Phytoplankton adaptations to low temperatures include cold adapted enzymes, specific membrane lipid composition, and cold shock and antifreeze proteins [2]. Young, Goldman [3] showed that Antarctic diatoms have a high Rubisco content, relative to temperate diatoms, that allows them to partially compensate for low catalytic rates at low temperatures. Physiological studies of ecologically important psychrophilic diatoms [4–13] generally show that polar diatoms respond to changes in irradiance using mechanisms similar to their temperate cousins: by enhancing non-photochemical quenching (NPQ) and by adjusting pigment composition and photosynthetic parameters. Physiological differences among polar diatoms and between polar diatoms and *Phaeocystis* were used to explain their distribution in the environment. Polar phytoplankton have relatively low growth rates (<0.6 d^-1^), low maximum photosynthesis rates (P_max_), low light saturation parameters for growth (K_E_), low light saturation parameters for photosynthesis (E_K_) and low C:N ratios in comparison to temperate species [14]. These characteristics are mainly related to low temperature. Acclimation to low temperatures appears similar to acclimation to high irradiance in *Fragilariopsis cylindrus* with higher NPQ, up-regulation of the *psbA* gene and up-regulation of high-light fucoxanthin Chl *a*/c-binding proteins (FCPs) that are involved in energy dissipation [15]. A significant capacity for cyclic electron flow has also been demonstrated in the Antarctic diatom *Fragilariopsis cylindrus* [16], particularly under Fe limitation [7]. Natural communities of Antarctic diatoms have also shown very low respiration rates [16].

In addition to low temperature, the polar environment is characterized by very variable and extreme variations in irradiance and day length [17]. Several studies have highlighted the physiological adaptations of polar diatoms to extremely low light that can be encountered under the ice and snow during winter and spring [14,18–20]. Nevertheless, during the transition to summer, diatoms can experience strong increases in light dose due to long photoperiods and high solar angle-of-incidence [21]. In these conditions, the physiological strategies of diatoms may be drastically modified [20]. Diatoms have to cope with two major challenges: 1) growing at temperatures that limit enzyme kinetics, and 2) growing across a wide range of irradiance, even though their temperature-limited growth rate saturates at low irradiance. The aim of this paper is to describe in depth the physiological adaptations to this extreme environment. We chose *C*. *neogracilis*, a major bloom-forming Arctic strain that dominates the subsurface in open waters in summer [22,23] *i*.*e*. when water temperature is close to 0°C and where light dose can be very high (long photoperiod, high solar angle-of-incidence). *C*. *neogracilis* is particularly well adapted to low temperature since its growth rate at 0°C (almost one division per day) is among the highest measured in culture at such low temperature [14]. In this study, we describe in detail the regulation of several steps of photosynthesis (light absorption, photochemistry, oxygen production, carbon fixation) to decipher the underlying mechanisms of this fitness.

## 2. Materials and methods

### Algal cultures

Unialgal cultures of *C*. *neogracilis* (Roscoff Culture Collection RCC 2278), isolated during the Malina cruise in the Beaufort sea [23,24] were grown in semi-continuous cultures in pre-filtered f/2 medium [25] enriched with silicate. Culture conditions were maintained by diluting cultures once a day [26] and gently aerating through 0.3μm-pore-filters. Continuous illumination was provided by white fluorescent tubes at 10, 23, 50, 80, 150 and 400 μmol photon m^-2^ s^-1^ as measured using a QSL-100 quantum sensor (Biospherical Instruments, San Diego, CA, USA) placed in the culture vessel. Cultures were grown in a growth chamber at 0 or 5°C. Culture sampling was undertaken after cultures reached steady state (*sensu* MacIntyre and Cullen [26]), i.e. after a minimum of 10 cell generations under each growth conditions. We used daily measures of the culture growth rate (Table 1), cell diameter and chlorophyll *a* (chl*a*) per cell to monitor the acclimation of the culture to the growth conditions [27].

**Table 1:** Terminology.

Symbol	Definition	Units
μ	Growth rate	d^-1^
μ_max_	Temperature specific maximum growth rate	d^-1^
K_E_	Light saturation parameter for growth	μmol photon m^-2^ s^-1^
ā*	Chl*a* specific, spectrally averaged absorption coefficient weighted by the irradiance spectrum	m^2^ mg Chl*a*^-1^
σ_PSII_	Effective absorption cross sections of PSII	A^2^ photon^-1^
σ_OPT_	Optical absorption cross sections of PSII	A^2^ photon^-1^
E_K_^i^	Light saturation parameter of a given process i (C fixation, ETR, O_2_ production, NPQ)	μmol photon m^-2^ s^-1^
P^C^_m_	Carbon specific light saturated photosynthetic rate measured by ^14^C incubation	d^-1^
P^C^_e_	Carbon specific photosynthetic rate at growth irradiance measured by ^14^C incubation	d^-1^
α*	Chl *a* specific initial slope of the PE curves	mg C mg chl*a*^-1^ h^-1^ *(*μmol photon m^-2^ s^-1^)^-1^
Φ_m_	Maximum quantum yield of the photosystem 2 (PSII)	Dimensionless
Φ_PSII_	Realized quantum yield of charge separation at the PSII	Dimensionless
ETR_m_	Maximum electron transport rate at PSII	e^-^ PSII^-1^ s^-1^
ETR_e_	PSII specific electron transport rate at PSII at the growth irradiance	e^-^ PSII^-1^ s^-1^
NPQ	Non-photochemical quenching of fluorescence	Dimensionless
NPQ_max_	Maximum non-photochemical quenching of fluorescence	Dimensionless

Cultures in triplicate were then sampled over 3 consecutive days (at 10:00) (except at 10 μmol photon m^-2^ s^-1^, 2 days) for cell enumeration, HPLC pigment analysis, particulate C and N analysis, ^14^C incubations, O_2_ net production incubations, variable fluorescence determinations, particulate light absorption measurements and determination of Rubisco content (RbcL). Cells are acclimated (balanced growth) when internal adjustment of metabolic pathways has ceased [28]. Sampling over several days helps to ensure that equilibrium is reached and reinforces the characterization of the phenotype. We computed means and standard deviations from biological and temporal replicates (n = 6 or n = 7). ^14^C incubations and O_2_ net production incubations were not done at 23 and 150 μmol photon m^-2^ s^-1^. In this paper, the irradiances to which cultures were acclimated are called “growth irradiance” and the irradiances used in assays are called “incubation irradiance”.

### Cell number, C and N, pigments

*C*. *neogracilis* cells were counted and sized (equivalent spherical diameter) before and after culture dilution using a Beckman Multisizer 4 Coulter Counter. The concentrations of particulate C and N were determined daily. For particulate carbon and nitrogen, an aliquot of 10 mL of algal culture was filtered onto glass-fiber filters (Whathman GF/F 0.7μm, 25mm) pre-combusted at 500°C for 12 h. Filters were kept desiccated before elemental analysis with a CHN analyzer (2400 Series II CHNS⁄ O; Perkin Elmer, Norwalk, CT, USA). For pigment analysis, an aliquot of algal culture (5mL) was filtered onto glass-fiber filters (Whathman GF/F 0.7μm, 25mm), immediately flash-frozen in liquid nitrogen and stored at -80°C until analysis using the protocol described in Zapata, Rodriguez [29]. The xanthophyll de-epoxidation state (%) was calculated as Dt/(Dd + Dt)*100, where Dd is diadinoxanthin, the epoxidized form and Dt is diatoxanthin, the de-epoxidizedf [30].

### Light absorption

A dual beam spectrophotometer (Perkin Elmer, Lambda 850) equipped with an integrating sphere was used to determine the spectral values of the optical density (OD (λ)) of the cultures. Filtered culture medium was used as reference. The chlorophyll *a*-specific absorption coefficient (a* (λ) in m^2^ mg Chl*a*^-1^) was calculated as follows:

$${\text{a}}^{*}(\lambda )=\frac{2.3\cdot \text{A}(\lambda )}{l\cdot [\text{Chl}a]}$$ Eq 1

where A is the absorbance of the sample, *l* is the optical pathlength in the cuvette (m) and [Chl*a*] is the chlorophyll *a* concentration (mg m^-3^).

The Chl *a*-specific, spectrally averaged absorption coefficient weighted by the growth irradiance spectrum (a* in m^2^ mg Chl*a*^-1^) was calculated as:

$${\text{a}}^{*}=\frac{\int_{400}^{700}{\text{a}}^{*}(\lambda )\cdot E(\lambda )}{\int_{400}^{700}E(\lambda )}$$ Eq 2

where a*(λ) and E(λ) are the absorption coefficient and the growth irradiance at a given wavelength.

### ^14^C experiments

The relationship between the rate of carbon fixation and irradiance was determined according to Lewis and Smith [31]. A 50-mL culture sample was collected in the 3 replicate cultures, and inoculated with inorganic ^14^C (NaH_14_CO_3_, 2 μCi mL^-1^). To determine the total amount of bicarbonate added, three 20-μL aliquots of inoculated culture sample were added to 50 μL of an organic base (ethanolamine) and 6 mL of the scintillation cocktail (Ecolume) into glass scintillation vials. Then 1-mL aliquots of the inoculated culture sample were dispensed into twenty-eight 7-mL glass scintillation vials. The vials were cooled (0 or 5°C) and exposed to 28 different light levels (from 0 to 2200 μmol photon m^-2^ s^-1^ provided by independent LEDs (LUXEON Rebel, Philips lumileds) from the bottom. The PAR (μmol photon m^-2^ s^-1^) in each alveolus was measured before incubation with an irradiance meter (Biospherical QSL-100) equipped with a 4π spherical quantum sensor. After 20 min of incubation, culture aliquots were fixed with 50 μL of buffered formalin and then acidified (250μL of HCl 50%) under the fume hood for 3 hours in order to remove the excess inorganic carbon (JGOFS protocol, UNESCO 1994). Finally, 6 mL of scintillation cocktail were added to each vial prior to counting in the liquid scintillation counter (Tri-Card, PerkinElmer). The chlorophyll *a*-specific carbon fixation rate was finally computed according to Parsons, Maita [32].

### Active fluorescence experiment

Variable fluorescence measurements were made using a Fluorescence Induction and Relaxation (FIRe) fluorometer (Satlantic, Halifax, NS, Canada) that applies a saturating, single turnover flash (STF, 100μs) of blue light (455 nm, 60-nm bandwidth) to the incubated sample. The FIRe generates a fluorescence induction curve (fluorescence detected at 680 nm) that can be used to estimate the effective absorption cross section of photosystem 2 (σ_PSII_, A^2^ photon^-1^), the minimum and maximum rates of fluorescence after dark acclimation (F_0_ and F_M_, rel. units), the rate of fluorescence at any given level of actinic irradiance (F_S_, rel. units) and the maximum rate of fluorescence under saturating actinic irradiance (F_M_’, rel. units) using the FIReWORX algorithm (Pers. Comm. Audrey Barnett) and the flash lamp calibration provided by Satlantic [33]. σ_PSII_, F_0_, and F_M_ were measured on culture subsamples that were dark-acclimated for 20 min. We found that 20 min was sufficient to fully relax non-photochemical quenching of F_0_ and F_M_. F_S_ and F_M_’ were measured repeatedly on the same culture subsample after 2 min exposures under an increasing range of actinic light levels (blue light, 450 nm). Those Rapid Light Curves-RLCs allowed to estimate the PSII specific electron transport rate ETR and the non-photochemical quenching of fluorescence (NPQ) (see below).

We estimated the maximum quantum yield of PSII (Φ_m_) from 20 min dark acclimated cells and the realized quantum yield of charge separation at the PSII (Φ_PSII_) as follows:

$$\Phi_{\text{m}}=\frac{{\text{F}}_{\text{V}}}{{\text{F}}_{\text{M}}}=\frac{{\text{F}}_{\text{M}}-{\text{F}}_{0}}{{\text{F}}_{\text{M}}}$$ Eq 3

ΦPSII=FM'−FSFM' Eq 4

The PSII specific electron transport rate (ETR, e^-^ PSII^-1^ s^-1^) was estimated as follows [34]:

$$\text{ETR}=\sigma_{\text{PSII}}\cdot \frac{\Phi_{PSII}}{\Phi_{M}}\cdot \text{E}\cdot 6.022\;10^{-3}$$ Eq 5

Where E is the incubation irradiance (μmol photon m^-2^ s^-1^) and 6.022 10^−3^ is a constant to convert σ_PSII_ to m^2^ μmol photon^-1^ from A^2^ photon^-1^.

The non-photochemical quenching was calculated as follows:

$$\text{NPQ}=\frac{{\text{F}}_{\text{M}}-{\text{F}}_{\text{M}}{}^{\prime }}{{\text{F}}_{\text{M}}{}^{\prime }}$$ Eq 6

### O_2_ net production incubation

Algal suspensions were concentrated to 10^9^–10^10^ cells L^-1^ by filtration onto glass fiber filters (0.7μm, 25mm). 2 mL of the resulting suspensions were used for incubation. A water bath refrigerated (Lauda) Chlorolab 2 system with LS2 100W halogen lamp (Hansatech Instruments Ltd.) was used for the experimental set-up. Light intensity was modified using neutral density filters (Thorlabs Inc.). A magnetic stirrer assured homogeneous cell and O_2_ distribution in the incubation chamber without inducing damage to the cells [35,36]. Calibration and measurements were performed at growth temperature. The system was calibrated every sampling day against 35g L^-1^ NaCl air-equilibrated and zero-oxygen solutions, the latter obtained by N_2_ sparging. Samples were placed into the chamber for five minutes to allow for temperature equilibration and to prevent recording any residual photosynthetic O_2_ net production. The dark respiration rate was measured first followed by photosynthesis measurements with progressively increasing light intensities, from 0 to 1000 μmol photon m^-2^ s^-1^ for 2–3 minutes to reach an oxygen evolution pseudo-steady state [37].

#### Data analysis

The initial slope and the maximum value of the rate versus E curves (^14^C fixation, O_2_ net production, ETR, NPQ) were estimated by fitting the equation of Platt, Gallegos [38] (with the photoinhibition parameter β) to the experimental rate and PAR values. The light-saturation parameter was obtained by dividing the maximum rate by the initial slope of the curve. Values of PAR from the different incubation devices were converted to growth chamber equivalent PAR (Growth irradiance, E) to account for spectral differences between the growth chamber and the incubation device’s light source (E_i_) using:

$$E=E_{i}\frac{\int_{400}^{700}a^{*}(\lambda )E_{i}(\lambda )}{\int_{400}^{700}a^{*}(\lambda )E(\lambda )}\frac{\int_{400}^{700}E(\lambda )}{\int_{400}^{700}E_{i}(\lambda )}$$ Eq 7

All the irradiances (both incubation and growth irradiances) presented in the manuscript are converted to growth chamber equivalent PAR.

### Protein analyses

For RbcL quantitation, 30 mL of each culture was harvested onto glass fibre filters (Whatman GF/F 0.7 μm pore size). Filters were flash-frozen in liquid nitrogen and stored at -80°C. Protein extractions were performed using the FastPrep-24 and bead lysing “matrix D” (MP Biomedicals), using 4 cycles of 60 s at 6.5 m s^-1^ in 750 μL of 1X extraction buffer (Agrisera). The supernatant was assayed using a detergent compatible (DC) assay kit against BGG standard (Bio-Rad), then equalized volumes containing 0.25 μg of denatured total protein containing 1x sample buffer (Invitrogen) and 50 mM DTT were loaded onto a 4–12% Bis Tris SDS-PAGE gel (Invitrogen). Each gel had a 5-point quantitation curve using RbcL (www.agrisera.se).

Proteins were separated via electrophoresis at 200 V then transferred to polyvinylidene difluoride (PVDF) membranes at 30 V. Membranes were blocked for 1 h in 2% _w/v_ ECL blocking agent (GE Healthcare) dissolved in TBS-T (Tris, 20 mM; NaCl, 137 mM; Tween-20, 0.1% _v/v_), then incubated in 1:20,000 rabbit polyclonal anti-RbcL antibody for 1 h (Agrisera, AS15-2955) and finally in 1:20,000 goat anti-rabbit IgG HRP conjugated antibody (Agrisera, AS09 602) for 1 h. Membranes were rinsed with TBS-T solution five times after each antibody incubation. Chemiluminscent images were obtained using ECL Ultra reagent (Lumigen, TMA-100) and a VersaDoc CCD imager (Bio-Rad). Band densities for samples were determined against the standard curve (Agrisera, AS15 2955S) using the ImageLab software (v 4.0, Biorad).

### Statistical tests

To test for differences between temperatures with regard to physiological characteristics we used a one-way ANCOVA model with irradiance as a covariate. The use of the covariate in the model allows a statistical control for the effects of irradiance to evaluate the temperature effect on the physiological parameters. Following a significant treatment effect, Tukey’s multiple comparison method was used to compare temperatures. The normality assumption was verified using Shapiro–Wilk statistics. Data analyses were performed using the Sigma Plot 12.5. We also tested differences between means using t-test.

## 3. Results

### Growth versus light curves

**Fig 1:**
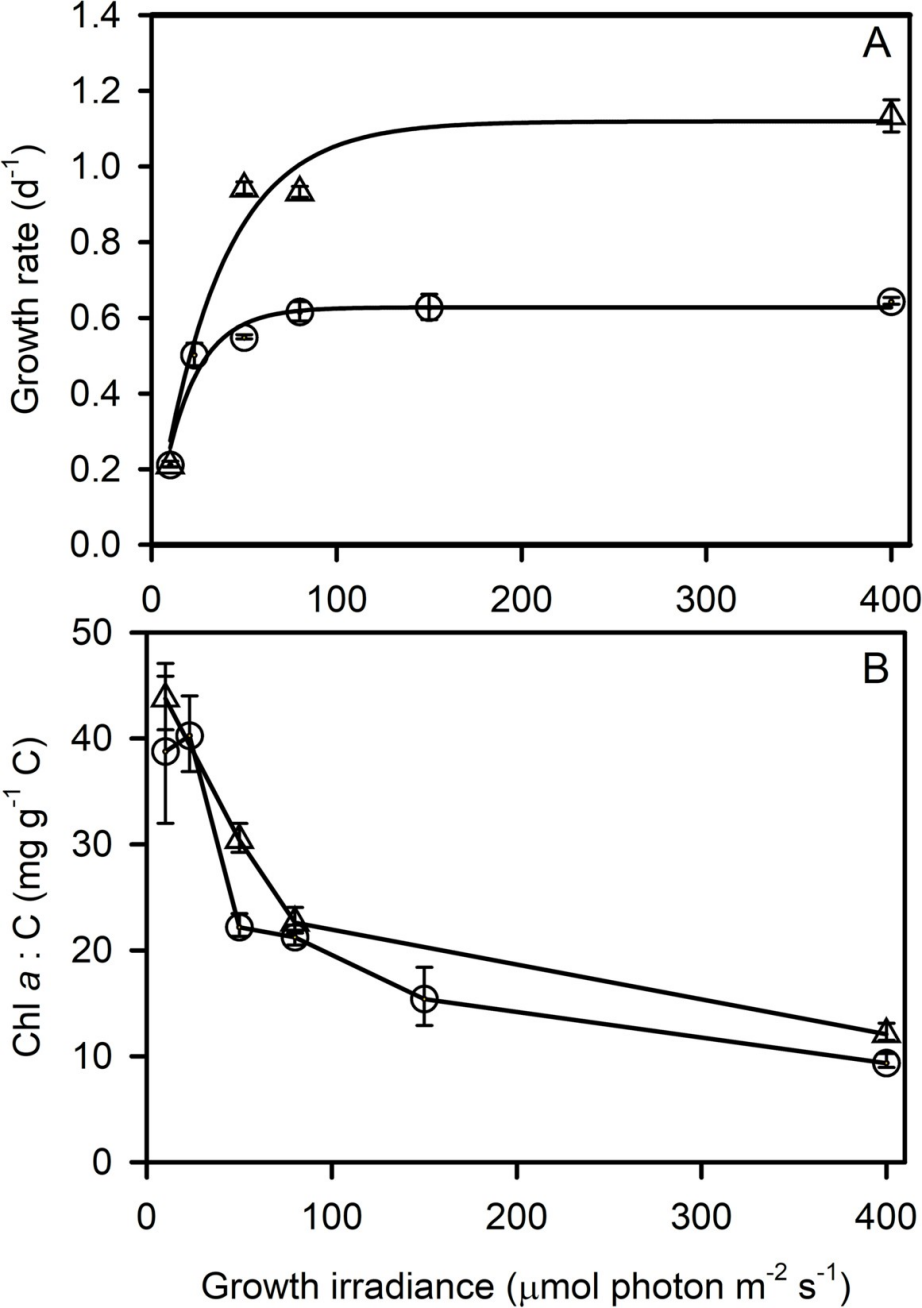
(A) Growth rate of *C*. *neogracilis* cultures versus growth irradiance at 0°C (circles) and 5°C (triangles). A Poisson function was fitted to the data and gives an estimate of μ_max_ of 0.63 d^-1^ and a K_E_ of 19 μmol photon m^-2^ s^-1^ at 0°C and a μ_max_ of 1.1 d^-1^ and a K_E_ of 35 μmol photon m^-2^ s^-1^ at 5°C. (B) Chl *a*: C ratio of *C*. *neogracilis* cells versus growth irradiance at 0°C (circles) and 5°C (triangles). In (A) each data point is the mean growth rate of 3 cultures measured each day over 10 consecutive days. In (B), each data point is the mean of 3 cultures measured each day over 3 consecutive days (23, 50, 80, 150, 400 μmol photon m^-2^ s^-1^) or 2 days (10 μmol photon m^-2^ s^-1^). Error bars represent standard deviations.

Fig 1A shows the growth-irradiance curves of *C*. *neogracilis* at 0°C and 5°C under nutrient repletion. A fit with a Poisson function [39] gives an estimate of the temperature specific maximum growth rate (μ_max_) of 1.1 d^-1^ and 0.63 d^-1^ and a light saturation parameter for growth (K_E_) of 35 and 19 μmol photon m^-2^ s^-1^ at 5 and 0°C, respectively. K_E_ delimits the boundary between growth-limiting and growth-saturating irradiances. The high growth rates at such low temperatures and the low K_E_ clearly show that this species is adapted to low light and low temperature. The ability to maintain high growth rates across a large range of irradiances with no photoinhibition of growth is particularly spectacular here, even under irradiance up to 20 times larger than K_E_, at such low temperatures.

Cell size increased with growth irradiance at both temperatures and mean cell volume was 42% larger at 0°C than at 5°C (ANCOVA, P<0.001) (S1 Fig). To track changes in pigment content, we therefore chose to normalize Chl *a* to particulate organic carbon (C) (see alternative normalisations in S2 Fig) as previously reported [39,40]. Modification of the Chl *a*: C ratio in response to growth irradiance (Fig 1B) was a prominent photoacclimation response in *C*. *neogracilis*. We observed a ≈3.5-fold decrease in Chl *a*: C between 10 and 400 μmol photon m^-2^ s^-1^. Surprisingly, Chl *a*: C did not significantly vary with growth temperature (ANCOVA, P = 0.9). We also measured the chlorophyll specific absorption coefficient (ā*, S3A Fig) and found insignificant differences between 0 and 5°C (ANCOVA, P = 0.7). These results suggest very different strategies in energy allocation at 5°C versus 0°C since cells grew at different growth rates while absorbing almost the same amount of photons per unit chlorophyll *a*.

### Carbon fixation

**Fig 2:**
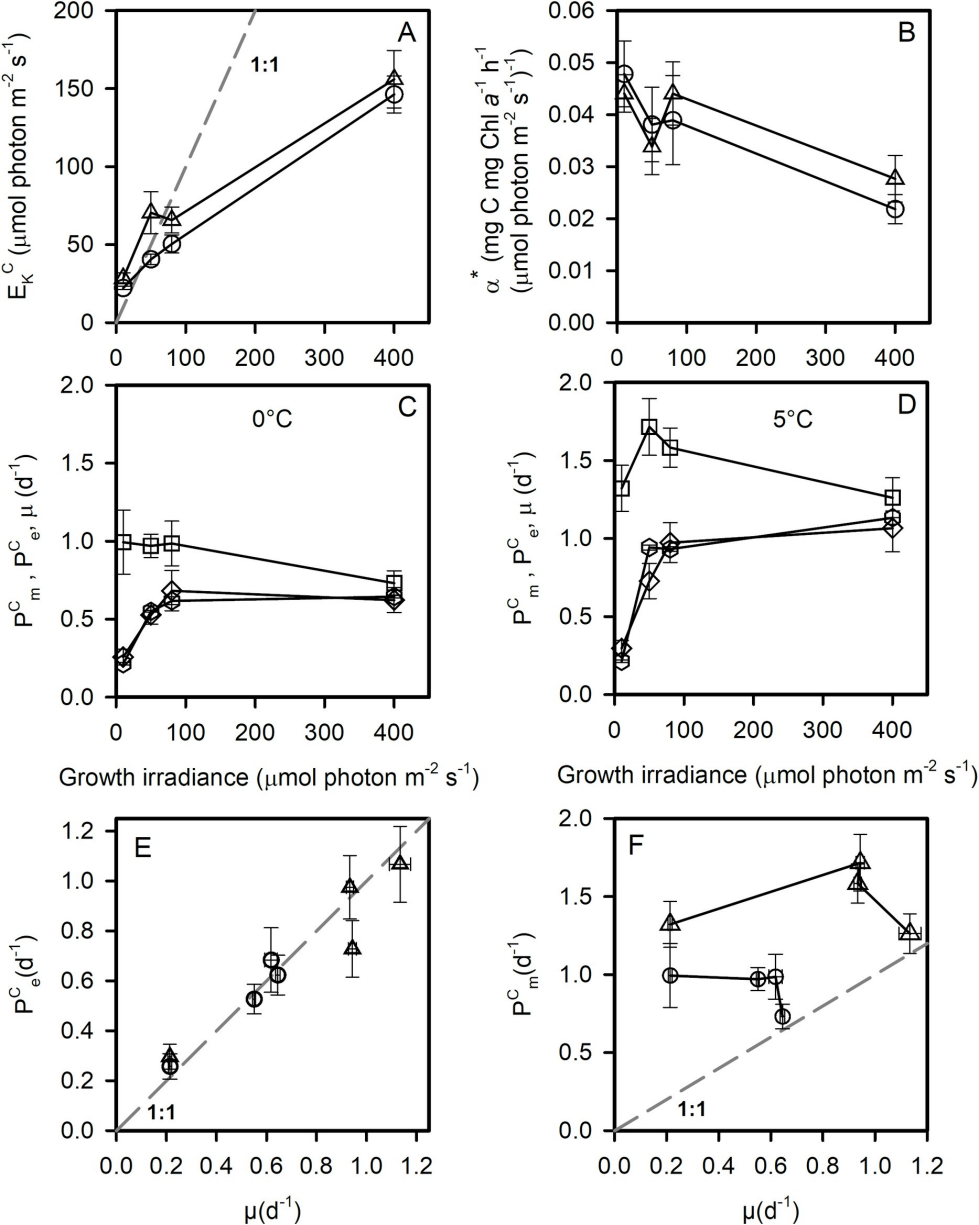
Photoacclimation of carbon fixation. E_K_^C^ (A), and α* (B) versus growth irradiance at 0°C (circles) and 5°C (triangles). P^C^_m_ (squares), P^C^_e_ (diamonds) and μ (hexagons) versus growth irradiance at 0°C (C) and 5°C (D). P^C^_e_ versus μ (E) and P^C^_m_ versus μ at 0°C (circles) and 5°C (triangle). Each data point is the mean of 3 cultures measured each day during 3 consecutive days (50, 80, 400 μmol photon m^-2^ s^-1^) or 2 days (10 μmol photon m^-2^ s^-1^). In A, E, F, the dotted lines represent the 1:1 line Error bars represent standard deviations.

Carbon fixation rates were obtained by measuring ^14^C incorporation into organic matter after 20 min incubations (S4 Fig). We fitted the data with the model of Platt, Gallegos [38] to obtain the light-saturated rate of carbon fixation normalized to particulate organic carbon (P^C^_m_), the Chl *a*-specific initial slope of the PE curve (α*) and the light saturation parameter (E_K_^C^) of the fixation rate *vs* irradiance curve, at each growth condition (Fig 2, alternative normalizations: S5 Fig). E_K_^C^ represents the irradiance at which carbon fixation begins to saturate in a given growth condition. It was linearly related to the growth irradiance (Fig 2A). According to the calculated linear regression equations, the growth irradiance at which E = E_K_^C^ was 58 ± 17 and 31 ± 4 μmol photon m^-2^ s^-1^ at 5 and 0°C, respectively. Carbon fixation by cells acclimated at 5°C saturated at higher irradiance than 0°C acclimated cells. The Chl *a*-specific initial slope of the PE curves (α*) decreased with increasing growth irradiance but was not significantly affected by growth temperature (Fig 2B).

The carbon-specific light-saturated rates of photosynthesis (P^C^_m_), the carbon-specific rates of photosynthesis at the growth irradiance (P^C^_e_) and the growth rates (μ) are plotted in Fig 2C (0°C) and 2D (5°C). Across all the growth conditions tested, we observed that P^C^_e_ was equal to μ (Fig 2E). P^C^_m_ did not increase with increasing growth irradiance and even decreased at 0°C. In most of the phytoplankton species studied, P^C^_m_remains almost constant across irradiances [39,40]. At 400 μmol photon m^-2^ s^-1^, P^C^_m_ was close to P^C^_e_ and μ. In these growth conditions, carbon fixation was thus completely saturated (Fig 2F). At both temperatures, under low irradiance, there was large apparent excess capacity for carbon fixation over achieved growth (Fig 2C, 2D and 2F).

### Rubisco

**Fig 3:**
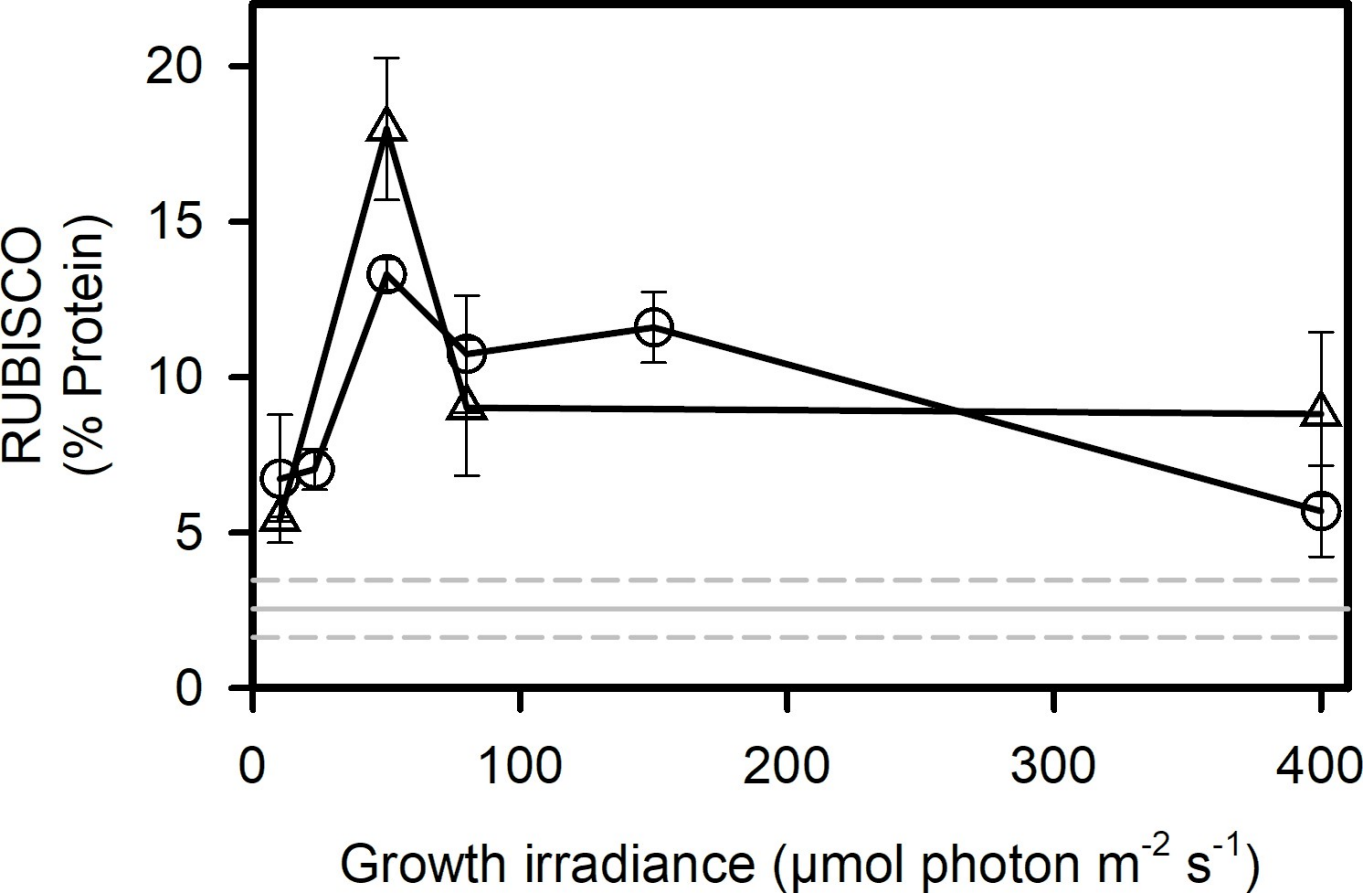
Photoacclimation of the Rubisco content. Rubisco content versus growth irradiance at 0°C (circles) and 5°C (triangles). The grey line represents the mean rubisco content measured in various mesophilic diatoms (*Thalassiosira weissflogii*, *Thalassiosira oceanica*, *Skeletonema costatum*, *Chaetoceros muelleri*, *Phaeodactylum tricornutum* from Losh et al. (2013)). Dashed lines represent standard deviations. Each data point is the mean of 3 cultures measured each day during 3 consecutive days (23, 50, 80, 150, 400 μmol photon m^-2^ s^-1^) or 2 days (10 μmol photon m^-2^ s^-1^). Error bars represent standard deviations.

In order to understand the role of dark reactions in the limitation of photosynthesis and growth, we quantified the amount of Rubisco in cells at each growth condition. Fig 3 shows that cells acclimated to 5 and 0°C had similar Rubisco contents. The Rubisco content peaked at 50 μmol photon m^-2^ s^-1^, *i*.*e*. around the irradiance at which growth became light-saturated. Rubisco content increased with growth irradiance in light-limited conditions and decreased in light-saturated conditions at both temperatures. Although the dark reactions of photosynthesis were probably limiting at high irradiance, cells did not compensate by increasing Rubisco content. Rubisco contents measured in this study (5–18% of total protein) are higher than the majority of the values reported for mesophilic diatoms [41–43] (see grey line in Fig 3) but accord well with those of psychrophilic diatoms (17% of total protein) [3].

### PSII electron transport rate (ETR), non-Photochemical Quenching (NPQ) and xanthophyll cycle pigments

Curves of ETR and NPQ versus actinic light were highly dependent on the culture acclimation light and temperature (S6 and S7 Figs). We were not able to saturate NPQ at the highest growth irradiances (150 μmol photon m^-2^ s^-1^ at 0°C and 400 μmol photon m^-2^ s^-1^ at both temperatures). Except for these points, ETR and NPQ increased with growth irradiance and decreased with growth temperature.

**Fig 4:**
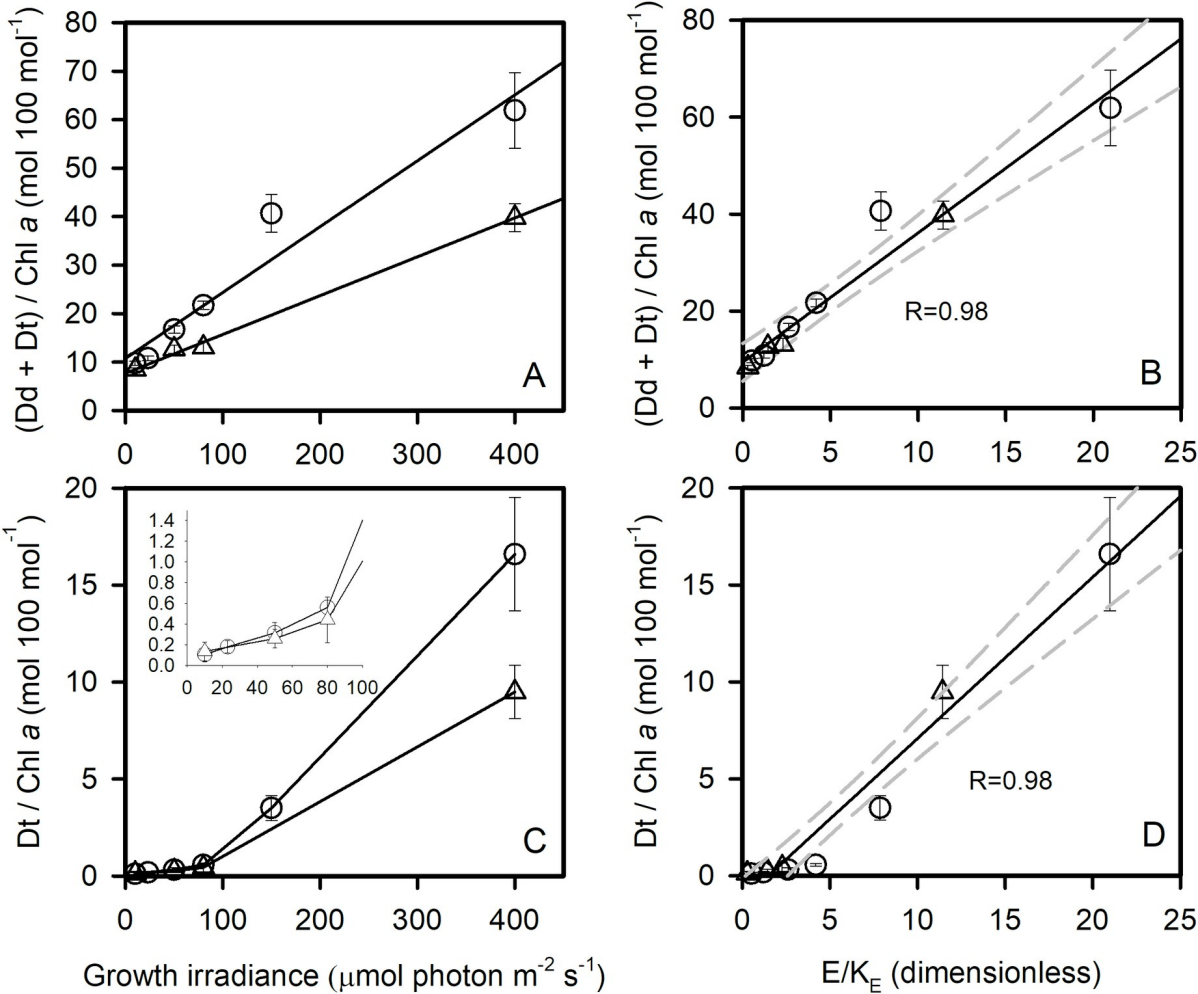
Effect of the acclimation irradiance on the xanthophyll cycle of *C*. *neogracilis*. (Dt +Dd)/Chl *a* (A) and Dt / Chl *a* (C) versus growth irradiance at 0°C (circles) and 5°C (triangles). (Dt +Dd) / Chl *a* (B) and Dt / Chl *a* (D) versus E/K_E_ at 0°C (circles) and 5°C (triangles). In C an insert shows an enlargement of low irradiances. Each data point is the mean of 3 cultures measured each day during 3 consecutive days (23, 50, 80, 150, 400 μmol photon m^-2^ s^-1^) or 2 days (10 μmol photon m^-2^ s^-1^). Error bars represent standard deviations.

The amount of diadinoxanthin (Dd) and diatoxanthin (Dt) (Fig 4A) increased with growth irradiance at both temperatures. The slope of the increase was significantly lower at 5°C than at 0°C (ANCOVA, Equal Slopes Test failed, P< 0.05). At the highest irradiances, the amount of xanthophyll pigments was very high. Dt / Chl *a* increased with growth irradiance and difference between temperatures was significant at 400 μmol photon m^-2^ s^-1^ (t-test, P<0.05). To consider the energy pressure in the cell at 0 and 5°C, we normalized the growth irradiance by K_E_. Fig 4B and 4D show that the relationships between E/K_E_ and the amount of xanthophyll pigments are not significantly different at 0 and 5°C (ANCOVA, Equal Slopes Tests passed, P> 0.05)

Relationships were found between the amount of Dd + Dt and E_K_^NPQ^ (S8A Fig) and NPQ_max_ (S8B Fig). As mentioned earlier, at 150 and 400 μmol photon m^-2^ s^-1^, we did not reach NPQ_max_ and consequently the evaluation of NPQ parameters (E_K_^NPQ^, NPQ_max_) is probably erroneous at those growth irradiances. Nevertheless, our results strongly suggest that cell’s energy balance (E/K_E_) control NPQ capacity through the control of the amount of xanthophyll pigments (Figs 4B and 4D and S8).

### Comparison of saturation irradiances (E_K_)

**Fig 5:**
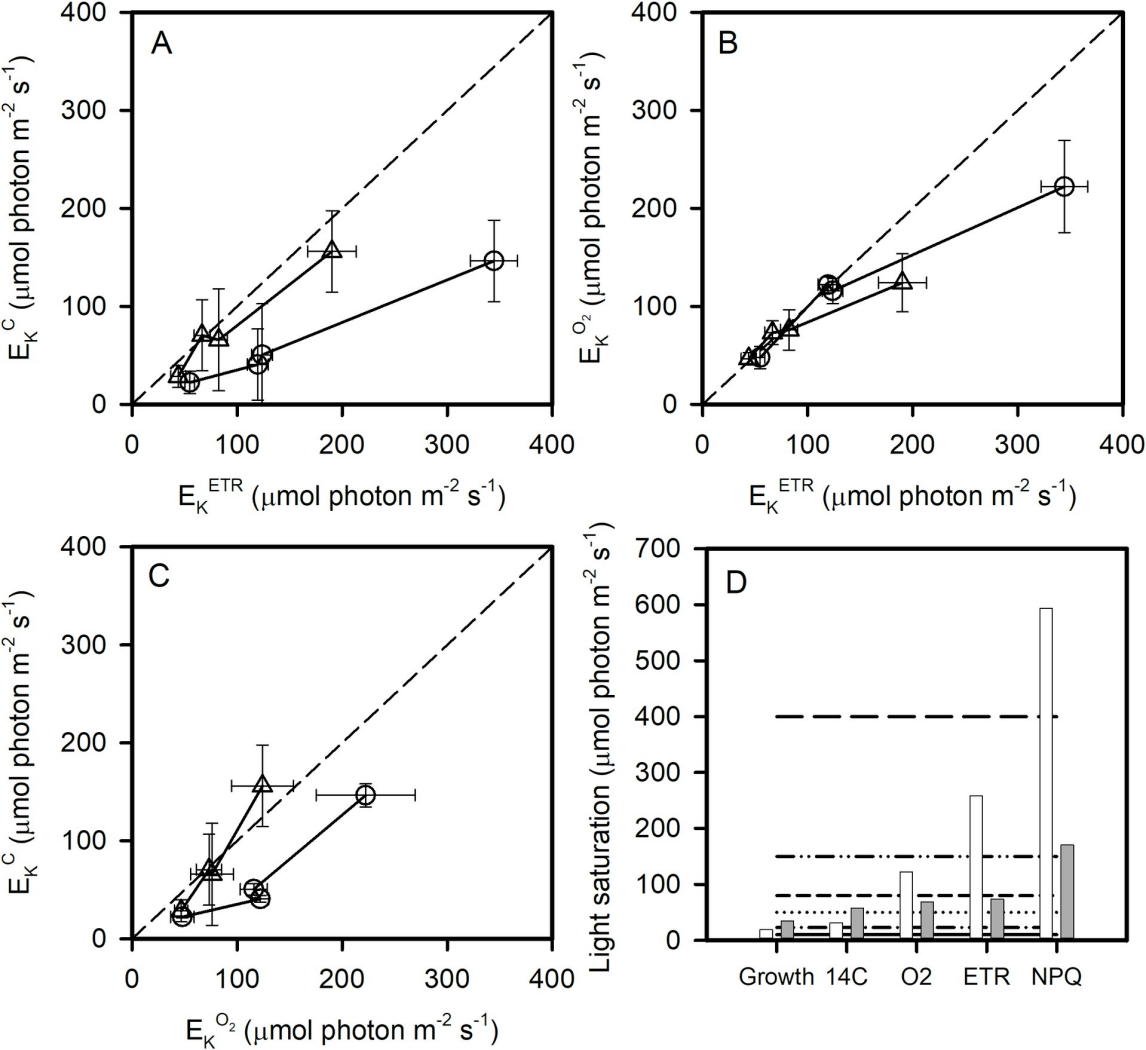
E_K_^C^ versus E_K_^ETR^ (A), E_K_^O^_2_ versus E_K_^ETR^ (B) and E_K_^C^ versus E_K_^O^_2_ (C) at 0°C (circles) and 5°C (triangles). In A, B, C, the dotted lines represent the 1:1 line. The growth irradiance at which growth rate (μ), carbon fixation (^14^C), O_2_ net production, ETR and NPQ saturate (E = E_K_^i^) is presented in D at 0°C (white bars) and 5°C (grey bars). In D, horizontal lines indicate growth irradiances (10, 23, 50, 80, 150, 400 μmol photon m^-2^ s^-1^).

As for ^14^C light curves, we fitted the ETR, NPQ, O_2_ production *vs* E data (S4, S6, S7 and S9 Figs) with the model of Platt, Gallegos [38] to estimate the threshold irradiance (E_K_^ETR^, E_K_^NPQ^ E_K_^O^_2_, μmol photon m^-2^ s^-1^) at which ETR, NPQ and O_2_ begin to saturate in relation to conditions (Fig 5). We found that, at both temperatures, when acclimated to low growth irradiance (10 μmol photon m^-2^ s^-1^), cellular carbon fixation and ETR saturate at similar incubation irradiances (Fig 5A). At 0°C in cells acclimated to higher irradiances, cellular carbon fixation saturates at a much lower incubation irradiance than does ETR (Fig 5A). Thus, at irradiances above E_K_^C^, although electron production is still increasing, carbon fixation is saturated, so electrons are flowing from PSII to acceptors other than CO_2_. In contrast at 5°C, the relationship between E_K_^C^ and E_K_^ETR^ is completely different with almost equal E_K_ for ETR and for carbon fixation across all growth irradiances, with the possible exception of the highest irradiances where carbon fixation saturates before ETR.

The comparison of E_K_^ETR^ and E_K_^O^_2_ gives additional insights on the cellular strategy in balancing energy availability and requirements (Fig 5B). For cells acclimated to low irradiances (10 μmol photon m^-2^ s^-1^), O_2_ net production and ETR saturate at the same incubation irradiance at both 5°C and 0°C. For cells acclimated to intermediate irradiances (50 and 80 μmol photon m^-2^ s^-1^), O_2_ net production and ETR also saturate at the same incubation irradiance, which is lower at 5°C than at 0°C. For high irradiance acclimated cells, measured O_2_ net production saturates at lower incubation irradiance than ETR.

The comparison of E_K_^C^ and E_K_^O2^ shows important differences between temperatures (Fig 5C). For cells acclimated to low irradiances (10 μmol photon m^-2^ s^-1^), O_2_ net production and carbon fixation saturate at around the same incubation irradiance at both 0°C and 5°C. At 0°C and for cells acclimated to higher irradiances, O_2_ net production saturates at higher irradiances than carbon fixation. This uncoupling shows that the electrons produced at PSII that are not used to reduce O_2_, are not all directed to carbon reduction and can be used in other processes. At 5°C, carbon fixation and O_2_ net production saturate at the same irradiance. This demonstrates that, at 0°C only, cells acclimated to moderate and high irradiances can use alternative electron acceptors (other than CO_2_ and O_2_) for electrons produced at PSII.

Fig 5D shows the estimated growth irradiances at which a given process saturates (growth, carbon fixation, O_2_ net production, ETR, NPQ) *i*.*e*. at E = E_K_^i^. These growth irradiances were computed from the linear regressions that are fitted to the relationship E_K_^i^ = f(E). Fig 5D illustrates that at a given acclimation irradiance some processes may be saturated and others not. Fig 5D also shows that O_2_ net production, ETR and NPQ saturation are strongly controlled by temperature.

## 4. Discussion

### Growth at low temperature

*C*. *neogracilis* is able to grow at almost a doubling per day at 0°C and 1.6 doublings per day at 5°C under continuous illumination, which is comparable to mesophilic diatom growth rates [44]. The rate-limiting step of C fixation is the first enzyme of the Calvin cycle *i*.*e*. Rubisco, especially at low temperature [3]. Rubisco content in *C*. *neogracilis* was high, in comparison to mesophilic diatoms. This strongly suggests that this psychrophilic species compensates for the slow Rubisco catalytic rates at low temperature by having a large Rubisco content. Our findings agree with previous studies which showed high Rubisco content and low activity in Antarctic diatoms [3] and psychrophilic green algae [45]. It remains unclear, however, if increasing Rubisco content at low temperature is an acclimation or an adaptation strategy. We showed that Rubisco content does not change with growth temperature between 5 and 0°C. So, over this range of temperature, *C*. *neogracilis* does not seem to acclimate to temperature by changing Rubisco content. This is in agreement with previous results [15] which showed no difference in the expression of the gene encoding the Rubisco large subunit in *Fragilariopsis cylindrus* at -1°C and 7°C. Cells may have reached a maximum Rubisco content at 5°C and cannot increase it further when temperature decreases. Indeed, *C*. *neogracilis* invests a huge amount of organic carbon, a possible limiting product for growth at low temperature, in proteins and may not be able to invest more, as previously suggested for Antarctic diatoms [3].

### Photo acclimation at low temperature

The growth rate of *C*. *neogracilis* saturates at low irradiance, which is a common characteristic of polar species [14]. In spite of this low K_E_, cells are able to maintain growth at much higher irradiances without any impact on growth rate nor on photosynthesis rates. We observed decreases in ETR at high incubation irradiance in a few environmental conditions (at 5°C in low light acclimated cells) but no decreases in carbon fixation or O_2_ net production rates (S4, S6 and S9 Figs). This shows that cells successfully mitigate potential effects of photoinhibition particularly by decreasing Chl *a* content and by enhancing NPQ, which is a classic pattern in phytoplankton acclimation to high irradiance [39,46]. Xanthophyll pigment content were higher at 0°C, which is consistent with the higher capacity for NPQ (Figs 4 and S8). The potential for photochemistry is also higher at 0°C (S6 Fig). This is in line with previous finding who suggested that photochemical acclimation to low temperature mimics acclimation to high irradiance [15,47,48]. As previously recognized [49], photoacclimation of pigments is accomplished through mechanisms that ‘integrate’ irradiance and temperature (and nutrients) through the combined effects of down-stream reactions relative to the photon flux. The validity of this concept is confirmed by the relationship between the amount of xanthophyll pigment ((Dd +Dt)/Chl *a*) and the growth irradiance corrected by K_E_ (Fig 4). This shows that energy pressure (E/K_E_), controlled by both temperature and irradiance, governed photoprotective capacity.

Except for the highest irradiances (400 μmol photon m^-2^ s^-1^), short-term carbon fixation was not totally saturated (P^C^_m_ >> P^C^_e_, Fig 2C and 2D), showing that Rubisco activity was not the rate limiting process in cell growth. Downstream reactions may be responsible for limiting growth such as, for example, the export of carbohydrates from the chloroplast which may also limit processes in plants at low temperatures [50]. We observed a surprising decrease in Rubisco content at high light, showing that cells allocated fewer resources into dark reactions in light-saturated conditions. Rubisco/Cell is generally not affected by growth irradiance [51]. The decrease of Rubisco content observed in our study at high irradiance may be the result of a limitation of resources (organic carbon and nitrogen) to support Rubisco accumulation. At high irradiance, the need for more proteins associated with photoprotection (*e*.*g*. PSII repair cycle [43], alternative electron cycles) may lead to a depletion of the chloroplastic biosynthetic resources needed to maintain or increase Rubisco content.

The decrease in Rubisco content with growth irradiance in light saturated cells may be responsible for the observed decrease in P^C^_m_. Decreases in P^C^_m_ with increasing irradiance have been observed in very few studies [39,52]. Anning, MacIntyre [52] observed a decrease in P^C^_m_ in *Skeletonema costatum* at high irradiance (1200 μmol photon m^-2^ s^-1^) and suggested that an increase in C reserves (lipid, carbohydrates) might decrease P^C^_m_ at high irradiance. The absence of any increase in C:N ratio with increasing irradiance (S1A Fig) suggests, however, no accumulation of carbon reserves at saturating irradiance. They also suggested that the control of P^C^_m_ shifts from enzymes of the Calvin cycle in low-light cells to the photosynthetic electron transfer chain in high-light cells. It is also probably not the case in the present study since electron transport saturated at much higher irradiance than carbon fixation (Fig 5A).

### Alternative electron pathways

We found that P^C^_e_ and μ are almost equal at all temperatures and irradiances tested. This implies that in these growth conditions, the 20 min ^14^C incorporation method provides a measure of the net carbon-specific production rate. It suggests that 1- any transient carbon pool has an average lifetime that is shorter than 20 min, or2- that only a small proportion of the fixed carbon is rapidly re-oxidized via respiratory processes. We recently investigated the lifetime of newly fixed carbon in another polar diatom species (*T*. *gravida*,unpublished) by decreasing the incubation time down to 2 min and observed no increase in the measured carbon fixation rate, suggesting no short-term oxidation of the newly fixed carbon. However, phytoplankton cells must use carbon-dependent pathways to produce ATP and reducing power to fuel a high growth rate, especially in a disturbed environment with changing L/D cycle, irradiance or temperature. Indeed, in most photoautotrophs, a significant proportion of the CO_2_ that is fixed into organic carbon (gross carbon production, GPP^C^) does not contribute to biomass (NPP^C^, net primary production) and is re-oxidized rapidly to regenerate ATP and NADPH [53] in order to fuel cell needs. The transient carbon pool, which can be very large (up to 63% of GPP^C^, see [54–57] is oxidized for either mitochondrial ATP production (O_2_-consuming pathways) or catabolism that provides electrons for carbon reduction (ex: non-O_2_ consuming oxidative pentose phosphate pathway) [54,55]. In *C*. *neogracilis*, if organic carbon oxidation is low, alternative electron and energy flows are necessary to supply the metabolic needs of the cell for nutrient assimilation, maintenance, and for synthesis of molecules that are more reduced than Glyceraldehyde-3-Phosphate, the initial product of the Calvin-Benson cycle. Low temperatures, by drastically limiting the dark phase of photosynthesis, may be an environmental constraint that favors alternative “carbon free” pathways of energy and reductant production in the cell. It is interesting to note that these measurements were carried out in a very simplified environment, *i*.*e*., under continuous light, after complete acclimation to stable growth conditions, which is very far from what algae experience in the natural environment. It is likely that the use of organic carbon as energy source and reducing power is much more crucial in the more variable natural environment (*e*.*g*. at night).

Photosynthetic parameters measured by ^14^C incorporation, O_2_ net production and variable fluorescence reflect different processes that are controlled differently by growth conditions. The comparisons of the E_K_ for carbon fixation, O_2_ net production and ETR provides evidence that reducing power produced by photosynthetic processes has numerous, quantitatively significant alternative fates in addition to its role in reducing CO_2_. The uncoupling between E_K_^C^, E_K_^ETR^ and E_K_^O^_2_ indicates a capacity for alternative electron flows (AEF). Low irradiance acclimated cells do not have the instantaneous capacity for alternative electron flows and thus are not able to divert electrons from carbon fixation even when subjected to high measurement irradiances. At moderate growth irradiances and even more certainly at high irradiance (400 μmol photon m^-2^ s^-1^), carbon fixation is saturated and alternative electron flows probably occur. Numerous authors have observed uncoupling between measured carbon fixation, O_2_ net production and electron transport rates, particularly at high irradiances [58–60]. Polar diatoms seem to have particularly high AEFs [61]. Recent studies have examined the processes that uncouple rates of CO_2_ assimilation and photosynthetic electron transport [62–65]. In the present study we showed that the ability to divert electrons from carbon fixation and the pathways used were not only dependent on instantaneous environmental conditions but also on the growth history (acclimation) of the cell.

We observed high potential uncoupling between electrons leaving PSII and measured O_2_ net production in cells acclimated to high irradiance. Similarly, in 3 Antarctic diatoms (*Fragilariopsis cylindrus*, *Pseudo-nitzschia subcurvata* and *Chaetoceros sp*.) decoupling between measured O_2_ net production and ETR was observed beyond E_K_ [12]. This decoupling is often interpreted as the result of cyclic electron flow (CEF) around PSII [7,66,67]. It can also be due to O_2_ re-consumption. The nature of the oxidases involved in those water-to-water cycles is unknown. These respiration processes may occur in the chloroplast (e.g. Melher reaction, PTOX) or in the mitochondria (e.g cytochrome pathway, cyanide resistant alternative pathway) [62,63,68,69]. Photorespiration should be relatively low at such low temperatures as Rubisco specificity for CO_2_ and CO_2_/O_2_ relative solubility in water increase with decreasing temperature [16,70,71]. Interestingly, our data show that temperature does not seem to influence these alternative electron flows.

We also observed uncoupling between measured saturation of O_2_ production and C fixation at low temperature only. It suggests that cells acclimated to low temperatures (0°C) are able to use alternative acceptors other than O_2_ for electrons produced at PSII. Direct use of reducing power (NADPH) and export out of the chloroplast (via for example Malate oxaloacetate transporters) is a classic feature in photosynthetic organisms [72]. NADPH may be involved in nutrient assimilation or in the reduction of organic molecules (e.g. fatty acids, nutrients). The direct use of reducing power has already been suggested in highly N-limited phytoplankton cells [56,57]. CEF around PSI can also be responsible for the observed uncoupling between O_2_ production and C fixation. CEF around PSI was probably very active in the Antarctic diatom *F*. *cylindrus* and in natural Antarctic diatom communities [16]., ATP production via the CEF around PSI is much less sensitive to low temperature than the multiple enzymatic reactions of the Krebs cycle and subsequent oxidation pathways to produce ATP [16]. It agrees with the observation that in *C*. *neogracilis*, O_2_/C uncoupling is only observed under low temperature (0°C) under which, carbon fixation is probably highly constrained.

The very low organic carbon oxidation rates suggested in our study and observed in other polar species [16] also support the idea that organic carbon oxidation is not the main pathway that produces ATP and reducing power in polar phytoplankton cells. Low temperatures may be an environmental constraint that promotes “carbon free” pathways of energy and reductant flows in the cell.

## Supporting information

S1 Fig C/N ratio (A) and Cell mean diameter (B) versus growth irradiance at 0 (circles) and 5°C (triangles). Each data point is the mean of 3 cultures measured each day during 3 consecutive days (23, 50, 80, 150, 400 μmol photon m-2 s-1) or 2 days (10 μmol photon m-2 s-1). Error bars represent standard deviations.
(DOCX)

S2 Fig Nitrogen per cell (A) and biovolume (B), C per cell (C) and biovolume (D), Chl a per cell (E) and biovolume (F) versus growth irradiance at 0 (circles) and 5°C (triangles). Each data point is the mean of 3 cultures measured each day during 3 consecutive days (23, 50, 80, 150, 400 μmol photon m-2 s-1) or 2 days (10 μmol photon m-2 s-1). Error bars represent standard deviations.
(DOCX)

S3 Fig Acclimation of *C*. *neogracilis* absorption properties and photochemistry. Chl*a* specific, spectrally averaged absorption coefficient weighted by the irradiance spectrum (A), the effective absorption cross sections of PSII (σ_PSII_, solid lines) and the optical absorption cross sections of PSII (σ^OPT^_PSII_, dotted lines) (B), and the maximum quantum yield of charge separation (C) versus growth irradiance at 0°C (circles) and 5°C (triangles). Each data point is the mean of 3 cultures measured each day during 3 consecutive days (23, 50, 80, 150, 400 μmol quanta m^-2^ s^-1^) or 2 days (10 μmol quanta m^-2^ s^-1^). Error bars represent standard deviations.
(DOCX)

S4 Fig Photoacclimation of carbon fixation. Carbon fixation rate in cell acclimated to 10, 50, 80 and 400 μmol photon m^-2^ s^-1^ versus incubation irradiance at 0°C (A) and 5°C (B). Each data point is the mean of 3 cultures measured each day during 3 consecutive days (50, 80, 400 μmol quanta m^-2^ s^-1^) or 2 days (10 μmol quanta m^-2^ s^-1^). Error bars represent standard deviations.
(DOCX)

S5 Fig Photoacclimation of carbon fixation. P_m_^Cell^ (A), and P_m_* (B) versus growth irradiance at 0°C (circles) and 5°C (triangles). Each data point is the mean of 3 cultures measured each day during 3 consecutive days (50, 80, 400 μmol quanta m^-2^ s^-1^) or 2 days (10 μmol quanta m^-2^ s^-1^). Error bars represent standard deviations.
(DOCX)

S6 Fig Acclimation of *C*. *neogracilis* photochemistry. Absolute PSII electron transport rates versus incubation irradiance for cultures acclimated to various irradiance at 0°C (A) and 5°C (B). Each data point is the mean of 3 cultures measured each day during 3 consecutive days (23, 50, 80, 150, 400 μmol quanta m^-2^ s^-1^) or 2 days (10 μmol quanta m^-2^ s^-1^). Error bars represent standard deviations.
(DOCX)

S7 Fig Acclimation of *C*. *neogracilis* NPQ. NPQ versus incubation irradiance at various growth irradiances at 0°C (A) and 5°C (B). Each data point is the mean of 3 cultures measured each day during 3 consecutive days (23, 50, 80, 150, 400 μmol quanta m^-2^ s^-1^) or 2 days (10 μmol quanta m^-2^ s^-1^). Error bars represent standard deviations.
(DOCX)

S8 Fig EKNPQ (A) and NPQmax (B) versus (Dt +Dd)/Chl a at 0°C (circles) and 5°C (circles). In A and B a regression line was fitted on the whole dataset (both 0 and 5°C) with the exception of the datapoints corresponding to 150 and 400 μmol photon m-2 s-1 for which NPQmax and EKNPQ are underestimated (see text).
(DOCX)

S9 Fig Acclimation of *C*. *neogracilis* O_2_ net production. O_2_ net production rates versus incubation irradiance for cultures acclimated to various irradiance at 0°C (A) and 5°C (B). Each data point is the mean of 3 cultures measured each day during 3 consecutive days (23, 50, 80, 150, 400 μmol quanta m^-2^ s^-1^) or 2 days (10 μmol quanta m^-2^ s^-1^). Error bars represent standard deviations.
(DOCX)

S10 Fig Data points. Data from: “Photoacclimation of the polar diatom Chaetoceros neogracilis at low temperature” by Thomas Lacour, Jade Larivière, Joannie Ferland, Philippe-Israël Morin, Pierre-Luc Grondin, Natalie Donaher, Amanda Cockshutt, Douglas A Campbell, Marcel Babin.
(DOCX)
