# Supplementary material for: Photoacclimation of the polar diatom Chaetoceros neogracilis at low temperature
Source: PLoS One. 2022 Sep 20;17(9):e0272822. doi: 10.1371/journal.pone.0272822 (PMC9488821; doi:10.1371/journal.pone.0272822)
Supplement: S1 Fig — C/N ratio (A) and Cell mean diameter (B) versus growth irradiance at 0 (circles) and 5°C (triangles). Each data point is the mean of 3 cultures measured each day during 3 consecutive days (23, 50, 80, 150, 400 μmol photon m-2 s-1) or 2 days (10 μmol photon m-2 s-1). Error bars represent standard deviations. (DOCX) [file pone.0272822.s001.docx]

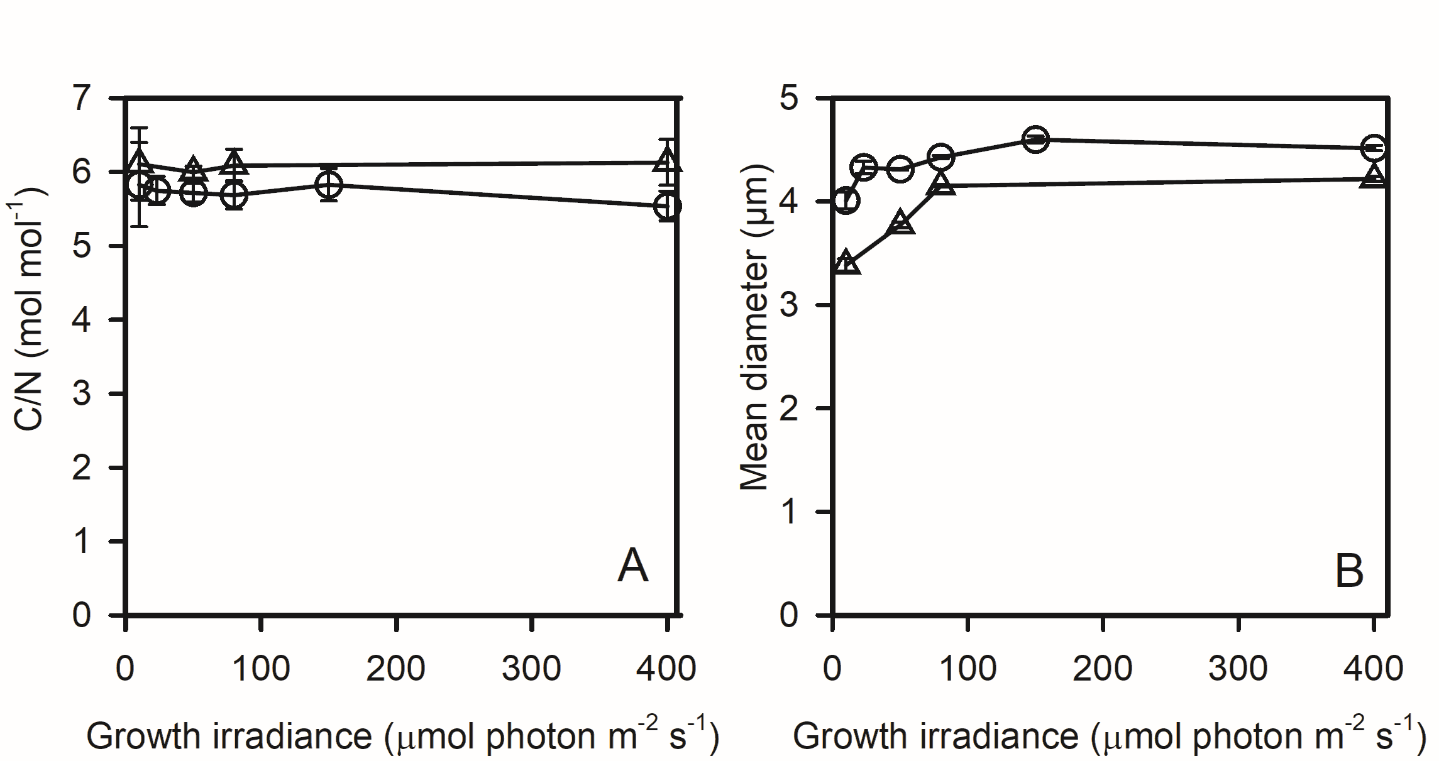


Figure S1: C/N ratio (A) and Cell mean diameter (B) versus growth irradiance at 0 (circles) and 5°C (triangles). Each data point is the mean of 3 cultures measured each day during 3 consecutive days (23, 50, 80, 150, 400 µmol photon m^-2^ s^-1^) or 2 days (10 µmol photon m^-2^ s^-1^). Error bars represent standard deviations.
