# Supplementary material for: Photoacclimation of the polar diatom Chaetoceros neogracilis at low temperature
Source: PLoS One. 2022 Sep 20;17(9):e0272822. doi: 10.1371/journal.pone.0272822 (PMC9488821; doi:10.1371/journal.pone.0272822)
Supplement: S3 Fig — Chla specific, spectrally averaged absorption coefficient weighted by the irradiance spectrum (A), the effective absorption cross sections of PSII (σPSII, solid lines) and the optical absorption cross sections of PSII (σOPTPSII, dotted lines) (B), and the maximum quantum yield of charge separation (C) versus growth irradiance at 0°C (circles) and 5°C (triangles). Each data point is the mean of 3 cultures measured each day during 3 consecutive days (23, 50, 80, 150, 400 μmol quanta m-2 s-1) or 2 days (10 μmol quanta m-2 s-1). Error bars represent standard deviations. (DOCX) [file pone.0272822.s003.docx]

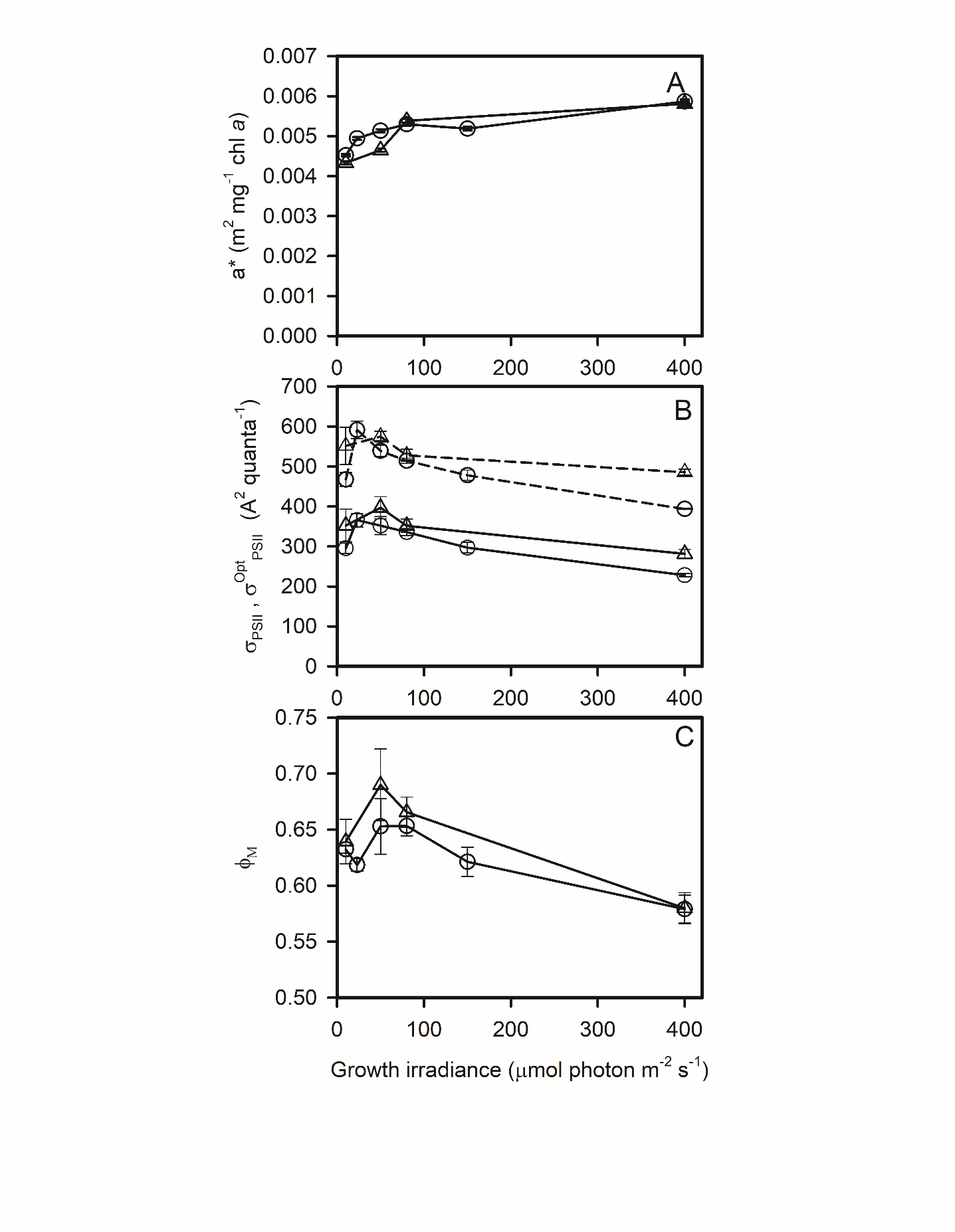


Figure S3: Acclimation of *C. neogracilis* absorption properties and photochemistry. Chl*a* specific, spectrally averaged absorption coefficient weighted by the irradiance spectrum (A), the effective absorption cross sections of PSII (σ_PSII_, solid lines) and the optical absorption cross sections of PSII (σ^OPT^_PSII_, dotted lines) (B), and the maximum quantum yield of charge separation (C) versus growth irradiance at 0°C (circles) and 5°C (triangles). Each data point is the mean of 3 cultures measured each day during 3 consecutive days (23, 50, 80, 150, 400 µmol quanta m^-2^ s^-1^) or 2 days (10 µmol quanta m^-2^ s^-1^). Error bars represent standard deviations.
