# Supplementary material for: Photoacclimation of the polar diatom Chaetoceros neogracilis at low temperature
Source: PLoS One. 2022 Sep 20;17(9):e0272822. doi: 10.1371/journal.pone.0272822 (PMC9488821; doi:10.1371/journal.pone.0272822)
Supplement: S8 Fig — EKNPQ (A) and NPQmax (B) versus (Dt +Dd)/Chl a at 0°C (circles) and 5°C (circles). In A and B a regression line was fitted on the whole dataset (both 0 and 5°C) with the exception of the datapoints corresponding to 150 and 400 μmol photon m-2 s-1 for which NPQmax and EKNPQ are underestimated (see text). (DOCX) [file pone.0272822.s008.docx]

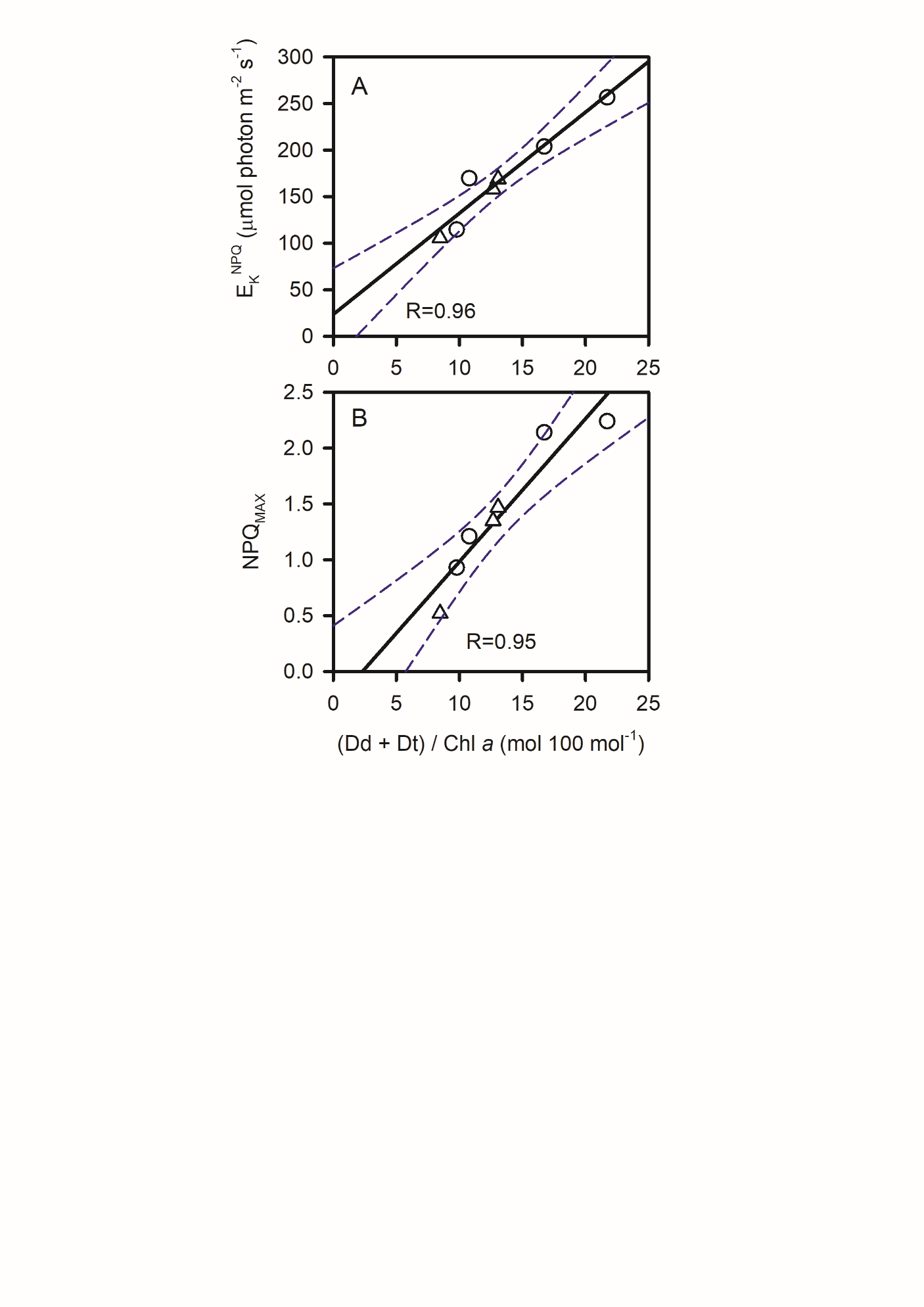


Figure S8: E_K_^NPQ^ (A) and NPQ_max_ (B) versus (Dt +Dd)/Chl *a* at 0°C (circles) and 5°C (circles). In A and B a regression line was fitted on the whole dataset (both 0 and 5°C) with the exception of the datapoints corresponding to 150 and 400 µmol photon m^-2^ s^-1^ for which NPQ_max_ and E_K_^NPQ^ are underestimated (see text).
