# Supplementary material for: Photoacclimation of the polar diatom Chaetoceros neogracilis at low temperature
Source: PLoS One. 2022 Sep 20;17(9):e0272822. doi: 10.1371/journal.pone.0272822 (PMC9488821; doi:10.1371/journal.pone.0272822)
Supplement: S9 Fig — O2 net production rates versus incubation irradiance for cultures acclimated to various irradiance at 0°C (A) and 5°C (B). Each data point is the mean of 3 cultures measured each day during 3 consecutive days (23, 50, 80, 150, 400 μmol quanta m-2 s-1) or 2 days (10 μmol quanta m-2 s-1). Error bars represent standard deviations. (DOCX) [file pone.0272822.s009.docx]

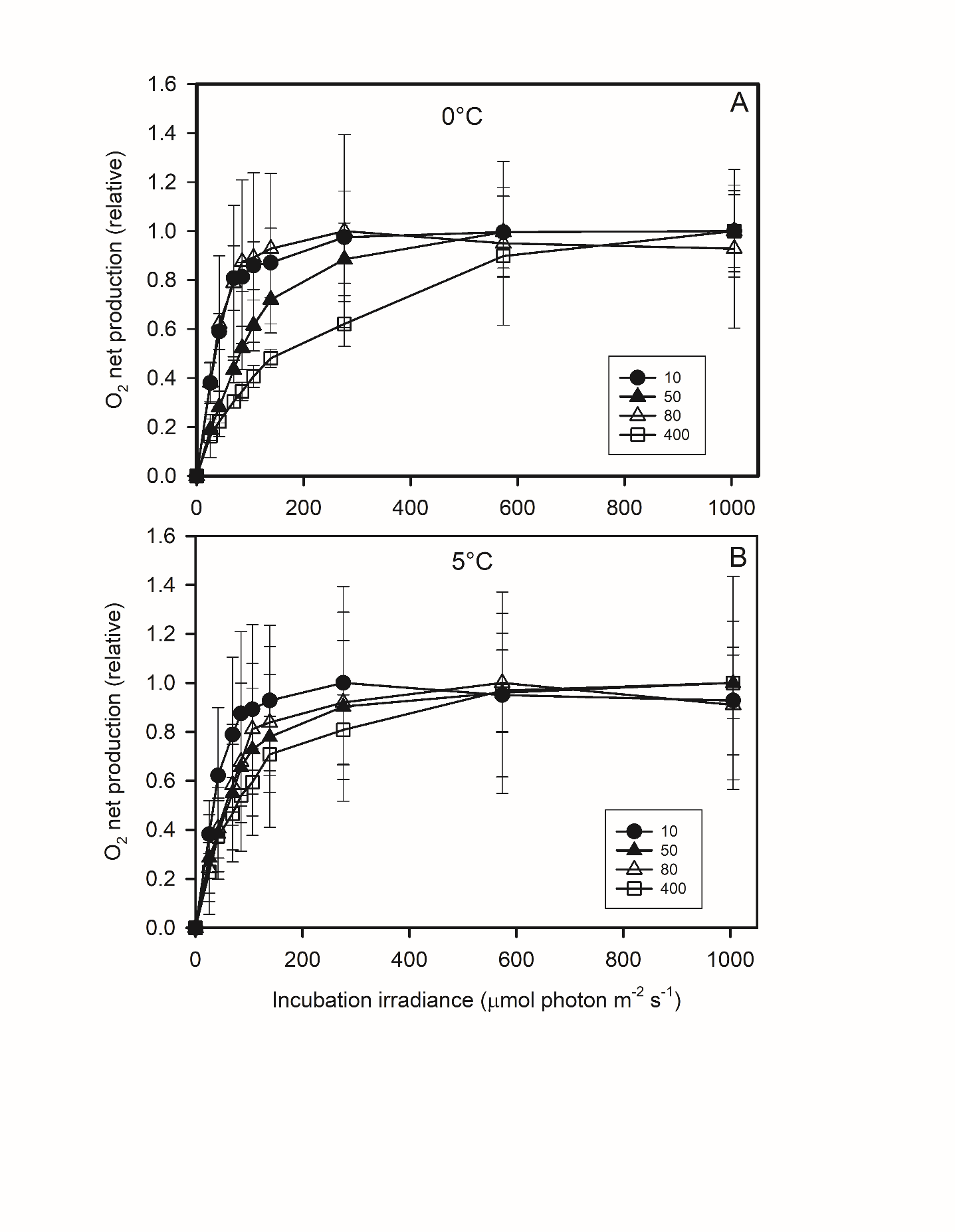


Figure S9: Acclimation of *C. neogracilis* O_2_ net production. O_2_ net production rates versus incubation irradiance for cultures acclimated to various irradiance at 0°C (A) and 5°C (B). Each data point is the mean of 3 cultures measured each day during 3 consecutive days (23, 50, 80, 150, 400 µmol quanta m^-2^ s^-1^) or 2 days (10 µmol quanta m^-2^ s^-1^). Error bars represent standard deviations.
