# Supplementary material for: Photoacclimation of the polar diatom Chaetoceros neogracilis at low temperature
Source: PLoS One. 2022 Sep 20;17(9):e0272822. doi: 10.1371/journal.pone.0272822 (PMC9488821; doi:10.1371/journal.pone.0272822)
Supplement: S10 Fig — Data from: “Photoacclimation of the polar diatom Chaetoceros neogracilis at low temperature” by Thomas Lacour, Jade Larivière, Joannie Ferland, Philippe-Israël Morin, Pierre-Luc Grondin, Natalie Donaher, Amanda Cockshutt, Douglas A Campbell, Marcel Babin. (DOCX) [file pone.0272822.s010.docx]

**Figure S10:** **Data points. Data from:** “**Photoacclimation of the polar diatom Chaetoceros neogracilis at low temperature” by** Thomas Lacour, Jade Larivière, Joannie Ferland, Philippe-Israël Morin, Pierre-Luc Grondin, Natalie Donaher, Amanda Cockshutt, Douglas A Campbell, Marcel Babin

|  | 0°C | | | 5°C | | |
| --- | --- | --- | --- | --- | --- | --- |
| **Growth rate (d-1)** | A | B | C | A | B | C |
| 10 | 0.21 | 0.22 | 0.22 | 0.21 | 0.22 | 0.21 |
| 23 | 0.51 | 0.53 | 0.47 |  |  |  |
| 50 | 0.55 | 0.54 | 0.55 | 0.93 | 0.94 | 0.96 |
| 80 | 0.59 | 0.63 | 0.63 | 0.94 | 0.92 | 0.94 |
| 150 | 0.60 | 0.62 | 0.67 |  |  |  |
| 400 | 0.65 | 0.65 | 0.64 | 1.12 | 1.10 | 1.18 |
| **Chla/C (mg g-1)** | A | B | C | A | B | C |
| 10 | 42.49 | 34.00 | 32.39 | 41.54 | 46.15 | 41.86 |
| 10 | 50.28 | 33.58 | 40.96 | 41.88 | 49.33 | 43.02 |
| 23 | 47.63 | 39.53 | 39.77 |  |  |  |
| 23 | 38.61 | 37.36 | 35.76 |  |  |  |
| 23 | 40.55 | 40.62 | 44.22 |  |  |  |
| 50 | 22.43 | 21.87 | 21.62 | 27.45 | 31.37 | 31.07 |
| 50 | 22.01 | 20.91 | 22.83 | 30.09 | 31.44 | 32.08 |
| 50 | 22.36 | 22.97 | 24.73 | 30.34 | 30.27 | 31.57 |
| 80 | 20.74 | 23.26 | 22.17 | 24.67 | 23.31 | 21.60 |
| 80 | 21.70 | 20.57 | 20.71 | 22.98 | 22.27 | 21.38 |
| 80 | 20.90 | 20.77 | 22.29 | 23.02 | 24.59 | 21.81 |
| 150 | 16.70 | 16.82 | 14.75 |  |  |  |
| 150 | 15.78 | 14.14 | 16.59 |  |  |  |
| 150 | 15.84 | 9.92 | 20.27 |  |  |  |
| 400 | 10.35 | 9.64 | 9.90 | 12.44 | 12.26 | 13.80 |
| 400 | 9.80 | 10.36 | 9.76 | 12.41 | 12.66 | 10.65 |
| 400 | 9.30 | 8.88 | 8.36 | 12.26 | 11.86 | 12.46 |
| **EkC (µmol photon m-2 s-1)** | A | B | C | A | B | C |
| 10 | 23.25 | 21.67 | 22.05 | 24.64 | 25.52 | 26.74 |
| 10 | 21.89 | 23.86 | 21.04 | 31.52 | 31.77 | 31.22 |
| 50 | 43.82 | 42.57 | 43.85 | 59.55 | 57.05 | 51.55 |
| 50 | 63.16 | 40.28 | 51.91 | 68.45 | 90.34 | 69.91 |
| 50 | 49.93 | 49.77 | 50.95 | 87.54 | 77.83 | 72.63 |
| 80 | 44.23 | 43.37 | 46.65 | 59.98 | 64.82 | 61.83 |
| 80 | 53.91 | 51.41 | 47.95 | 65.02 | 59.94 | 57.21 |
| 80 | 49.72 | 61.24 | 52.28 | 85.75 | 75.38 | 68.69 |
| 400 | 130.03 | 150.49 | 128.92 | 148.25 | 155.01 | 138.12 |
| 400 | 149.94 | 157.11 | 136.77 | 146.41 | 147.29 | 140.38 |
| 400 | 155.36 | 161.48 | 146.93 | 166.20 | 194.89 | 173.21 |
| **Pce (d-1)** | A | B | C | A | B | C |
| 10 | 0.288 | 0.221 | 0.222 | 0.24 | 0.34 | 0.33 |
| 10 | 0.342 | 0.211 | 0.261 | 0.28 | 0.24 | 0.35 |
| 50 | 0.598 | 0.594 | 0.581 | 0.74 | 0.72 | 0.89 |
| 50 | 0.451 | 0.542 | 0.555 | 0.69 | 0.64 | 0.94 |
| 50 | 0.459 | 0.468 | 0.495 | 0.67 | 0.64 | 0.63 |
| 80 | 0.8376 | 0.9219 | 0.7256 | 1.23 | 0.89 | 0.77 |
| 80 | 0.5976 | 0.5375 | 0.6913 | 1.01 | 1.03 | 1.01 |
| 80 | 0.5907 | 0.5661 | 0.6790 | 0.97 | 0.98 | 0.88 |
| 400 | 0.7404 | 0.7266 | 0.6939 | 0.86 | 1.22 | 1.16 |
| 400 | 0.5796 | 0.6272 | 0.6049 | 1.14 | 1.30 | 1.02 |
| 400 | 0.5683 | 0.5420 | 0.5244 | 0.94 | 0.89 | 1.08 |
| **PCm (d-1)** | A | B | C | A | B | C |
| 10 | 1.19 | 0.89 | 0.95 | 1.17 | 1.36 | 1.13 |
| 10 | 1.26 | 0.70 | 0.98 | 1.45 | 1.50 | 1.32 |
| 50 | 1.02 | 1.01 | 1.02 | 1.32 | 1.64 | 1.70 |
| 50 | 0.96 | 0.95 | 1.09 | 1.77 | 1.95 | 1.82 |
| 50 | 0.89 | 0.85 | 0.95 | 1.60 | 1.84 | 1.80 |
| 80 | 1.13 | 1.27 | 0.97 | 1.72 | 1.37 | 1.43 |
| 80 | 0.86 | 0.99 | 0.99 | 1.58 | 1.63 | 1.53 |
| 80 | 0.86 | 0.81 | 1.00 | 1.75 | 1.59 | 1.63 |
| 400 | 0.84 | 0.84 | 0.79 | 1.17 | 1.19 | 1.38 |
| 400 | 0.68 | 0.74 | 0.70 | 1.36 | 1.51 | 1.17 |
| 400 | 0.66 | 0.72 | 0.61 | 1.21 | 1.13 | 1.24 |
| **alpha*** | A | B | C | A | B | C |
| 10 | 0.050 | 0.050 | 0.055 | 0.048 | 0.048 | 0.042 |
| 10 | 0.048 | 0.036 | 0.048 | 0.046 | 0.040 | 0.041 |
| 50 | 0.043 | 0.045 | 0.045 | 0.034 | 0.038 | 0.044 |
| 50 | 0.029 | 0.047 | 0.038 | 0.036 | 0.029 | 0.034 |
| 50 | 0.033 | 0.031 | 0.031 | 0.025 | 0.033 | 0.033 |
| 80 | 0.0512 | 0.0523 | 0.0391 | 0.049 | 0.038 | 0.045 |
| 80 | 0.0307 | 0.0390 | 0.0414 | 0.044 | 0.051 | 0.052 |
| 80 | 0.0345 | 0.0264 | 0.0359 | 0.037 | 0.036 | 0.045 |
| 400 | 0.0261 | 0.0241 | 0.0257 | 0.026 | 0.026 | 0.030 |
| 400 | 0.0193 | 0.0188 | 0.0219 | 0.031 | 0.034 | 0.033 |
| 400 | 0.0191 | 0.0210 | 0.0207 | 0.025 | 0.020 | 0.024 |
| **Rub (%protein)** | A | B | C | A | B | C |
| 10 | 8.83 | 4.72 | 6.67 | 5.47 | 5.49 | 5.34 |
| 23 | 6.43 | 6.97 | 7.72 |  |  |  |
| 50 | 13.32 | 13.80 | 12.80 | 15.37 | 18.97 | 19.61 |
| 80 | 8.59 | 11.61 | 12.04 | 6.71 | 11.06 | 9.31 |
| 150 | 10.77 | 11.15 | 12.91 |  |  |  |
| 400 | 4.60 | 5.13 | 7.36 | 7.74 | 6.91 | 11.82 |
| **(Dd+Dt)/(100Chla)** | A | B | C | A | B | C |
| 10 | 10.44 | 10.12 | 9.49 | 9.00 | 8.70 | 8.36 |
| 10 | 9.80 | 9.25 | 9.57 | 8.38 | 8.36 | 7.98 |
| 23 | 9.70 | 10.58 | 10.80 |  |  |  |
| 23 | 11.35 | 11.33 | 10.73 |  |  |  |
| 23 | 10.92 | 11.04 | 10.65 |  |  |  |
| 50 | 17.29 | 17.63 | 15.81 | 13.22 | 12.00 | 10.87 |
| 50 | 15.83 | 17.36 | 16.72 | 13.59 | 12.91 | 13.60 |
| 50 | 16.04 | 17.63 | 16.26 | 12.34 | 12.52 | 13.00 |
| 80 | 21.64 | 21.95 | 20.48 | 13.92 | 15.26 | 14.72 |
| 80 | 22.75 | 20.61 | 21.77 | 12.12 | 12.81 | 12.68 |
| 80 | 21.37 | 21.95 | 22.96 | 12.73 | 12.25 | 11.20 |
| 150 | 41.91 | 42.78 | 37.52 |  |  |  |
| 150 | 39.86 | 40.25 | 40.31 |  |  |  |
| 150 | 35.23 | 49.15 | 38.88 |  |  |  |
| 400 | 45.22 | 57.25 | 58.47 | 41.17 | 45.34 | 42.80 |
| 400 | 64.09 | 71.88 | 68.90 | 38.53 | 38.86 | 39.45 |
| 400 | 61.54 | 65.72 | 63.95 | 39.13 | 37.35 | 35.80 |
| **Dt/100 Chla** |  |  |  |  |  |  |
| 10 | 0.02 | 0.13 | 0.12 | 0.22 | 0.12 | 0.25 |
| 10 | 0.11 | 0.05 | 0.21 | 0.08 | 0.15 | 0.00 |
| 23 | 0.25 | 0.12 | 0.23 |  |  |  |
| 23 | 0.22 | 0.19 | 0.16 |  |  |  |
| 23 | 0.16 | 0.20 | 0.07 |  |  |  |
| 50 | 0.28 | 0.33 | 0.26 | 0.19 | 0.16 | 0.17 |
| 50 | 0.22 | 0.21 | 0.47 | 0.32 | 0.26 | 0.42 |
| 50 | 0.32 | 0.50 | 0.26 | 0.22 | 0.26 | 0.32 |
| 80 | 0.56 | 0.65 | 0.45 | 0.38 | 0.17 | 0.23 |
| 80 | 0.54 | 0.62 | 0.59 | 0.86 | 0.69 | 0.44 |
| 80 | 0.45 | 0.61 | 0.58 | 0.46 | 0.34 | 0.40 |
| 150 | 3.27 | 3.61 | 2.85 |  |  |  |
| 150 | 3.53 | 4.01 | 3.53 |  |  |  |
| 150 | 2.58 | 4.78 | 3.39 |  |  |  |
| 400 | 10.61 | 14.72 | 17.90 | 8.49 | 11.05 | 9.33 |
| 400 | 15.22 | 20.15 | 19.48 | 9.90 | 10.68 | 11.28 |
| 400 | 15.63 | 18.31 | 17.31 | 9.08 | 8.52 | 7.12 |
